# Progress in Medicine

**Published:** 1897-08-21

**Authors:** 


### Progress in Medicine.

### DISEASES OF THE HEART AND CIRCULATION.

(*Concluded from page* 340).

**Theobromine.**—Huchard[23] says that theobromine is preferable to diuretin as a diuretic. The headache so often produced by theobromine is best avoided by progressive dosage, beginning with 24 grains a day. The drug has no direct action on the heart, but acts directly on the renal epithelium. It may be long-continued, for its action is not cumulative and but slightly toxic; its action is rapid and is produced from the first day of its administration. It succeeds in some cases where digitalis and caffein have failed; its diuresis is more rapid and as abundant and certain as that produced by digitalis, and more abundant and certain than that produced by caffein. It acts well in heart disease with dropsy, and in severe cases of cirrhosis of the liver it gives good results, especially when given with a milk diet.

**Calomel.**—Moldarescu[24] states that calomel is of great value in the treatment of cardiac disease. He gives as much as 8 to 10 grains a day, divided into six doses. Diuresis begins after the first few doses, and by the second or third day copious evacuations of the bowels take place. To prevent salivation he gives a chlorate of potassium and tannic acid mouth wash. He believes that the way in which calomel does good is by relieving the congestion of the liver and kidneys.

**Idiopathic Arterial Hypermyopathy.**—We owe to Savill[25] the differentiation of idiopathic arterial hypermyopathy from chronic Bright's disease and from atheroma, with which it has hitherto been confused. A hypertrophic increase of the middle muscular coats of the arteries has long been known in association with chronic renal affections, but it may occur as an idiopathic disease by no means infrequently in advanced life and in the prematurely old. The affection may continue for many years unrecognised as long as the consequent cardiac hypertrophy compensates for the increased peripheral resistance. The symptoms which result from heart failure are: (1) Vertigo, which may come on without warning, especially after assuming the erect posture, and which may be immediately followed by actual syncope or vomiting. This vertigo has to be distinguished from that of Menière's disease, petit mal, and cerebral tumour, which can, as a rule, be easily done by the associated symptoms and physical signs. (2) Paroxysmal dyspnœa, to be distinguished from that of emphysema, which is so common at that time of life by its paroxysmal character. (3) Headache, probably due, as in chronic Bright's disease, to increased arterial tension. (4) Cerebral hæmorrhage. The symptoms, then, are very much the same as in chronic Bright's disease with consecutive cardio-vascular disease; but it is to be differentiated during life by the absence of the characteristic changes in the urine of that disease, *i.e.*, increased quantity with diminished specific gravity, and if the case go on to death by the fact that the kidneys are found to be healthy. The arteries are uniformly thickened, readily to be distinguished from the thickening of atheroma by its uniform character, and absence of bead-like irregularities. The prognosis is a good deal better than that of cases of renal cirrhosis, for the progress of the disease is much slower, and even if cerebral hæmorrhage occurs the prognosis of that condition is better than when this occurs in renal cirrhosis. The main indication for treatment is to keep the vascular tension low, by calomel and saline purges, and if necessary rest in bed, whilst for the headache, giddiness, and dyspnœa nitro-glycerine is the sovereign remedy.

**Atheroma and Aneurism.**—Coats and Auld,[26] from an extended series of microscopical examinations, find that the primary lesion in atheroma is essentially one of the intima—an increase of the subendothelial connective tissue, the endothelium remaining unchanged. This newly-formed connective tissue is very prone to undergo degenerative changes, of which fatty degeneration is by far the most common; and, secondarily, to such degenerative changes calcareous infiltration is added—most commonly in the aorta, rarely in the smaller cerebral vessels. The media may undergo secondary degenerative changes, similar to those occurring in the patch itself, and almost invariably it becomes thinned owing to atrophy of the tissue elements, particularly of the elastic tissue. The histology of aneurismal dilatation of the arteries is exactly similar to that of atheroma. The authors point out that the aneurismal period is that where the period of atheroma overlaps that of severe physical exertion, and the causation is intimately associated with the thinning of the media which results from the primary process in the intima.

[23] Huchard, L'Union Méd., No. 11, 1896. [24] Moldarescu, Revue de Therapeut. Méd. Chir., June, 1896. [25] Savill, Brit. Med. Journ., Jan. 23, 1897. [26] Coats and Auld, Jour. of Path., vol. iv.

### DISEASE OF THE PHARYNX AND LARYNX.

**Pharynx.**—A curious case of exostosis of the pharyngeal vault is recorded by Lichtwitz.[1] It was removed accidentally with adenoid vegetations.

The exostosis was situated high up, and had the form of a triangular crest, measuring a centimètre in length and half a centimètre in height. It was composed of normal spongy bone, and had no intimate connection with the adenoids. Somewhat similar cases have been described by Zuckerkandl, Scheff, Helme, and Roth, but they are undoubtedly extremely rare. The exact nature of the abnormality is altogether obscure. The explanation which falls in best with the present instance is to be found in a remark by Zuckerkandl: "The anterior tubercle of the atlas, as well as the crest of the axis, are variable, being sometimes markedly developed. These physiological structures may simulate a tumour of the posterior pharyngeal wall." The pharyngeal tubercle may in this instance be the part involved, presenting an anomaly similar to that occurring in the tubercle of the atlas and axis. Lermoyez considered one of M. Helme's cases mentioned was an exaggerated rostrum on the sphenoid.

**Pharyngitis and Tonsillitis.**—Pakes[2] has examined upwards of 500 serum tubes inoculated from the throats of patients in the wards or out-patients of Guy's Hospital. In five cases the author found the bacillus of Friedländer, twice in pure cultivation, twice associated with the Klebs-Loeffler bacillus, and once with the staphylococcus aureus. Clinically, the cases were indistinguishable from mild subacute follicular tonsillitis, with slight constitutional disturbance. Thus Case 5 was a girl aged six. The tonsils were red and swollen, and a few plugs of whitish material were present. The temperature was 100 deg. Fahr. A pure culture of Friedländer's bacillus was found on the blood serum. The author refers to Nicolle and Hébert's published account of the occurrence of the pneumo-bacillus of Friedländer on the throat of patients suffering from tonsillitis, follicular tonsillitis, or membranous pharyngitis, when upwards of 1,600 serum tubes inoculated from the throats of patients were examined by them, and they found the pneumo-bacillus of Friedländer eight times, six times alone.

For the prevention of follicular tonsillitis, Maxson[3] advises a method which he has successfully employed for ten years. He states he has never known it to fail to afford a very complete immunity, even when a patient is subject to attacks every two or three weeks before treatment. The method consists in taking a doubled piece of silver wire, such as is used for sutures, and soldering its free ends to a large wire; then dipping the doubled silver loop into melted nitrate of silver, so as to make a small bead on its end, after withdrawing it from the melted silver salt. The silver wire is then bent half an inch from its distal end, so as to make an angle of 45 deg. This bead of caustic is inserted into each follicle to its bottom; the depth will vary from one-fourth to one-half an inch. One caustic bead will usually treat from one to two follicles. By means of several beads all the follicles may be treated. This treatment is undertaken at any time except when the tonsils are acutely inflamed. It requires one or two treatments effectually to prevent its return.

**New Growths.**—If we have to deal with a fibroma, which is of doubtful character, it is well to remove a portion, and submit to microscopic examination. For this purpose Horne[4] recently treated a piece of such tissue. The piece of tissue was treated by a method to which Dr. Kanthack has recently drawn attention. It was placed in a test-tube with water and boiled; boiling for a minute and a half sufficed for the size of the tissue. It was then ready for cutting on a freezing microtome. The sections were stained in hæmatin and mounted in the usual way, the whole process requiring from fifteen minutes to half an hour. The water being sterilised by boiling, the tissue could have been safely left corked in the test tube for 24 or 26 hours. Tissues hardened by this rapid method can be preserved for longer periods in alcohol or Müller's fluid, or treated by the paraffin method.

**Lymphangioma of the Tonsil.**—Hamilton[5] figures and describes a case in a young man, aged 22. The growth was pear-shaped, measuring three inches by three-quarters of an inch, by half an inch. The surface was pink in colour and perfectly smooth; soft and doughy in consistence. Microscopic examination revealed a large quantity of fat, together with lymphangiomatous tissue.

**Motor Innervation.**—In investigating the position of the vocal cords after section of the recurrent nerves, and the influence of the crico-thyroid muscle, Grossmann[6] found, as a result of sixty experiments, that after section of one recurrent the glottis contracts itself; after the second nerve has been cut through there is another contraction of the glottis; but after destruction of both nervi laryng. super. the glottis opened again. So the author says that one cannot exclude from the adduction of the glottis paralysis of the positives. He mentions, further, the theories of Semon, Krause, &c., with the remark that a real cadaveric position of the vocal cords never can be noticed in lifetime.

**Laryngeal Paralysis.**—A case well illustrating the slow progression of paralytic lesion in locomotor ataxy was recently shown by Dundas Grant[7] at the Laryngological Society. When seen two years before he had almost complete abductor paralysis, and the right side of the palate was paralysed, being drawn up to the left on phonation, there being no dimple on his right side. At the time he was shown his epiglottis was very pendulous, and he had no power of lifting it. On inspiration there was a very wide elliptical opening between the cords, between the apposed anterior fourth, and his vocal process on either side, the latter being separated by two or three millimetres. The outward movement of the cartilages of Santorini was very slight on phonation, the vocal processes coming close together, and the elliptical opening between the vocal cords became somewhat narrower. The sensibility of the larynx was diminished, his voice being monotone. Grant considered there was paralysis of the abductors, and both internal and external tensors. Cresswell Baker, *àpropos* of Grant's case, related a case of abductor paralysis in a tabetic patient, in which there was also hyperæsthesia with attacks of abductor spasm.

We may here draw attention to the case of tabes dorsalis, complicated by laryngeal crises with laryngeal vertigo, recorded by Tresilian.[8] The choking attacks were taken by the patient to be whooping cough.

[1] Journ. of Laryng., May, 1897. [2] Brit. Med. Journ., Mar. 20, 1897. [3] Journ. of Pract. Med., Dec., 1896. [4] Proc. of Laryngol. Soc. of Lond., Jan., 1897. [5] Absts. Journ. of Laryng., Mar., 1897. [6] K. K. Gesellschft. der Aerzte in Wien, Apr., 1897; Journ. of Laryng., June. [7] Trans. of Laryng. Soc. of Lond., Apr., 1897. [8] Journ. of Laryng., May, 1897.

